# Step Up: An Evaluation of Question-Behavior Effects in Diabetes Footcare in Middle-Aged and Older Adults

**DOI:** 10.1093/geroni/igaa057.1468

**Published:** 2020-12-16

**Authors:** Anna Robertson, Katherine Johanson, Allison Walden, Rebecca Ingram, Lauren Schneider, Leilani Feliciano

**Affiliations:** 1 University of Colorado, Colorado Springs, COLORADO SPRINGS, Colorado, United States; 2 University of Colorado, Colorado Springs, Colorado Springs, Colorado, United States; 3 UCCS, Colorado Springs, Colorado, United States

## Abstract

Type 2 diabetes mellitus (T2DM) is a major public health concern requiring engagement with specific self-care activities for improvement. Due to the increased risk for neuropathy, foot ulcers, and infections, engagement with certain footcare practices (e.g., inspecting feet and inside shoes, washing and drying between toes) is an important part of overall diabetes self-care. However, interventions targeting blood glucose monitoring, medication management, nutrition, and/or physical activity often precede footcare interventions. Question-Behavior Effects (QBEs), which describe the effects of behavior questioning on future engagement in that behavior, provide a unique framework for studying diabetes self-care. Although clinicians may not administer footcare interventions for people with T2DM, footcare behaviors are often still assessed (e.g., through questionnaires) in session and, consistent with QBEs, may improve through the repetition of questioning. The present study evaluated footcare QBEs (specifically, the number of days of foot inspection and cleaning in the past week) across 7 sessions of an at-home diabetes management program without intentional footcare intervention. Paired-samples t-tests (N = 78, ages 40-88 years) revealed significant improvements in the number of days of foot inspection and cleaning from intake to session 1 and intake to session 5. However, from intake to session 7, only significant improvements in foot inspection were detected. These findings reflect the potency of footcare QBEs in the absence of specific footcare interventions. They also reveal differential engagement in foot inspection and cleaning after an extended period of questioning, suggesting certain diabetes self-care behaviors may taper without the intentional introduction of intervention.

